# Hypoglycemia From Metastatic Meningeal Solitary Fibrous Tumor Occurring Over Two Decades After Initial Diagnosis

**DOI:** 10.1210/jcemcr/luad001

**Published:** 2023-02-01

**Authors:** John J Orrego, Joseph A Chorny

**Affiliations:** Department of Endocrinology and Metabolism, Colorado Permanente Medical Group, P.C., Denver, CO 80205, USA; Department of Pathology, Colorado Permanente Medical Group, P.C., Denver, CO 80239, USA

**Keywords:** non-islet cell tumor hypoglycemia, NICTH, paraneoplastic hypoglycemia, hemangiopericytoma, solitary fibrous tumor, SFT

## Abstract

Despite multiple intracranial and extracranial relapses associated with a widely metastatic meningeal solitary fibrous tumor (formerly classified as hemangiopericytoma), a 66-year-old type 2 diabetic man was first diagnosed with paraneoplastic hypoglycemia 23 years after the original diagnosis and 12 years after the onset of extracranial metastatic disease. An enlarging mass entirely replacing the left kidney measuring 11.6 × 10 × 28 cm, which had not been locally treated before, was considered to be the putative source of IGF-2 excess. The insulin-like effects of IGF-2 not only ameliorated his long-standing type 2 diabetes mellitus, but also caused spontaneous fasting hypoglycemia. The physiopathology, clinical manifestations, diagnostic approach, and treatment of non-islet cell tumor hypoglycemia are briefly discussed here. Palliative tumor debulking improved the hypoglycemia by day 11 after radiation therapy and glucose monitoring with continuous glucose monitoring system (Dexcom G6) facilitated the patient's management and gave him peace of mind.

Tumoral hypoglycemia is caused by islet cell tumors (insulinomas) and non-islet cell tumors. Non-islet cell tumor hypoglycemia (NICTH) is a rare paraneoplastic syndrome caused not only by ectopic tumoral secretion of incompletely processed forms of pro-insulin-growth factor-2 (pro-IGF-2), known as “big” IGF-2, but also by an increased amount of circulating free “mature” IGF-2 peptide. This condition has also been termed *IGF-2-oma* and is produced most frequently by certain mesenchymal and epithelial tumors [1]. The most common mesenchymal tumors associated with NICTH are pleural or extrapleural solitary fibrous tumors (SFTs), which may be benign or malignant. Meningeal SFTs are rare intracranial soft-tissue tumors that resemble meningiomas on clinical and radiological grounds. Until recently, these meningeal tumors that arise from pericytes in the walls of capillaries were known as hemangiopericytomas [2].

We describe a 66-year-old type 2 diabetic man with NICTH caused by a metastatic meningeal SFT, which initially ameliorated his long-standing type 2 diabetes mellitus, and subsequently caused spontaneous fasting hypoglycemia. The latter was first detected 23 years after the original tumor diagnosis and 12 years after the onset of extracranial metastatic disease.

## Case Presentation

A 43-year-old Caucasian man presenting with a generalized seizure in 1998 was found to have a 5.6 cm parasagittal meningeal hemangiopericytoma involving the right parietal lobe and extending up to the sagittal sinus. He underwent subtotal tumor removal followed by Gamma Knife radiosurgery.

He was diagnosed with type 2 diabetes mellitus in 2002, initially treated with sulfonylureas, and then switched to Humulin N 25 units before breakfast and 15 units before dinner and Humulin R 5 units before meals in 2015, and glargine 50 units once daily and Humalog 5 units before meals in 2017.

He had local tumor recurrences in 2005 and 2009 that were treated with Gamma Knife radiosurgery and Trilogy radiosurgery, respectively.

Extracranial metastatic disease manifested by a 13.5-cm right infiltrative kidney mass and 2 lytic bone lesions was first detected in March 2010. He underwent a right nephrectomy and palliative radiation therapy to both lytic lesions, one in the anterior left iliac bone and the other in the left sacrum.

Between 2010 and July 2021, he had multiple relapses that were treated with surgery (brain), radiofrequency ablation (liver and bone), cryoablation (bone, pancreas, and liver), microwave ablation (bone), stereotactic radiosurgery (bone), radiation therapy (pancreas, left lung, bone, and left pelvis), intravenous bisphosphonates and denosumab (bone), and systemic chemotherapy (sorafenib, sulfatinib, temozolomide with bevacizumab, and pazopanib) with no long-term response. His most recent palliative treatment was stereotactic ablative radiotherapy to a left pelvic mass in July 2021 (27 Gy in 3 fractions).

From 2014 to July 2018, while on insulin, his glycated hemoglobin (HbA1c) remained in the 6.3% to 8.5% range. Off insulin, his HbA1c gradually dropped from 5.8% to <4.2% in May 2020.

**Figure 1.**
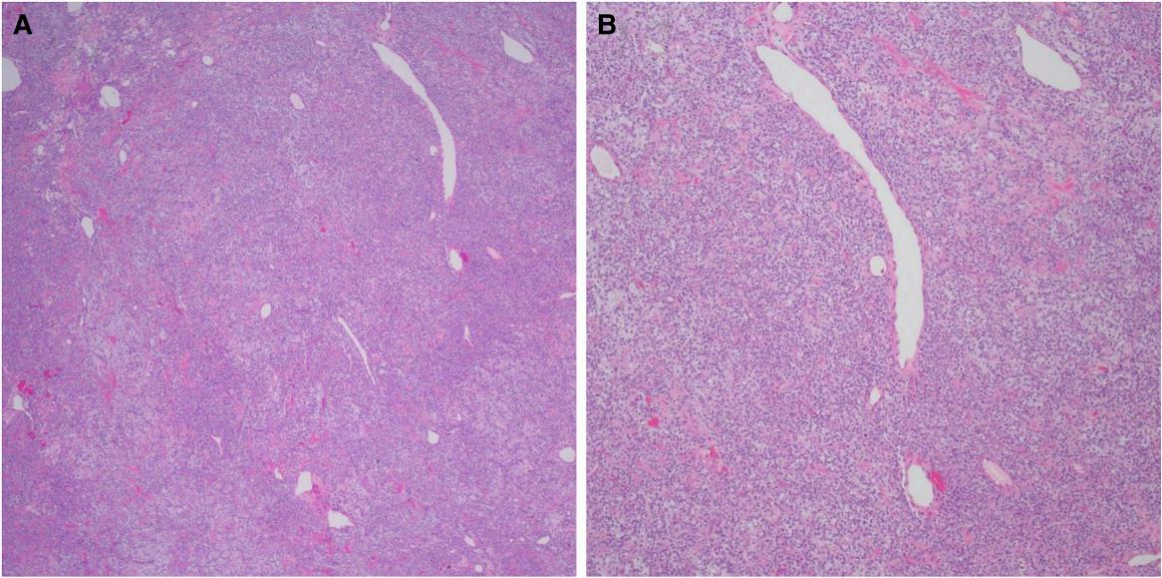
(A and B) Photomicrographs from this case of the metastatic SFT which is composed of uniform fibroblastic spindle cells in a “patternless pattern” with a staghorn vasculature and myxoid background (H&E, 20× and 40×).

In August 2018, excisional biopsy of left supraclavicular and left inguinal masses were consistent with metastatic malignant meningeal SFT (Fig. 1A and 1B). By immunohistochemistry, the tumor cells were positive for both CD34 and STAT6.

His renal function gradually declined after his right nephrectomy, and he was started on peritoneal dialysis in January 2021 and subsequently switched to hemodialysis.

### Diagnostic Assessment

The patient was admitted to the hospital in September 2021 for fasting hypoglycemia. He reported neuroglycopenic and adrenergic symptoms associated with capillary glucose levels in the 30 to 40 mg/dL range in the middle of the night, which resolved within 15 minutes after carbohydrate intake. He required intravenous and oral glucose during the hospital stay to avoid hypoglycemia. A brain magnetic resonance imaging (MRI) scan showed no evidence of recurrent or residual tumor. A computed tomography (CT) scan of his chest, abdomen, and pelvis revealed an enlarging 5 × 5.4 × 18.2-cm lobulated mass infiltrating the left kidney, and a new 6.9 × 5.3 cm central pelvic anterior mass. The left upper anterior subpleural nodule, the 3 enhancing capsular hepatic nodules, the multifocal pelvic, and L2 posterior vertebral body metastases, the 8-cm porta hepatis mass with central calcification, and the diffuse pancreatic nodularity were unchanged.

He was evaluated by endocrinology and non-islet cell tumor hypoglycemia (NICTH) was suspected and subsequently confirmed (Table 1) [3]. The IGF-2/IGF-1 ratio was 6.7, which is above the normal molar ratio of 3:1, but lower than the suggested diagnostic cutoff of 1:10. Since there is no commercially available assay, “big” IGF-2 was not measured.

**Table 1.** Patient's data and normal reference ranges, in conventional and SI units, respectively

	Glucose	Insulin	C-peptide	Proinsulin	β-hydroxybutyrate	GH	IGF-1	IGF-2	IGFBP-3
Patient data	37 mg/dL	<1.0 µU/mL	0.7 ng/mL	0.58 µU/mL	0.62 mg/dL	1.0 ng/mL	81 ng/mL	546 ng/mL	2.0 mg/L
Normal range	<55	<3.0	<0.8	<0.72	<28.1	<7.1	34-240	267-616	3.0-6.6
Patient data	2.05 mmol/L	<6.9 pmol/L	0.23 nmol/L	<4.0 pmol/L	0.06 mmol/L	2.25 pmol/L	10.6 nmol/L	1365 mmol/L	0.04 mmol/L
Normal range	<3.05	<20.8	<0.27	<5.0	<2.7	<16	4.5-31.4	642-1540	0.06-0.14

### Treatment

Diazoxide 200 mg twice daily and prednisone 30 mg daily were started and a Dexcom G6 continuous glucose monitor (CGM) was prescribed to facilitate the management of his hypoglycemia. Given persistent hypoglycemia, growth hormone (GH) therapy was entertained but palliative tumor debulking was recommended.

**Figure 2.**
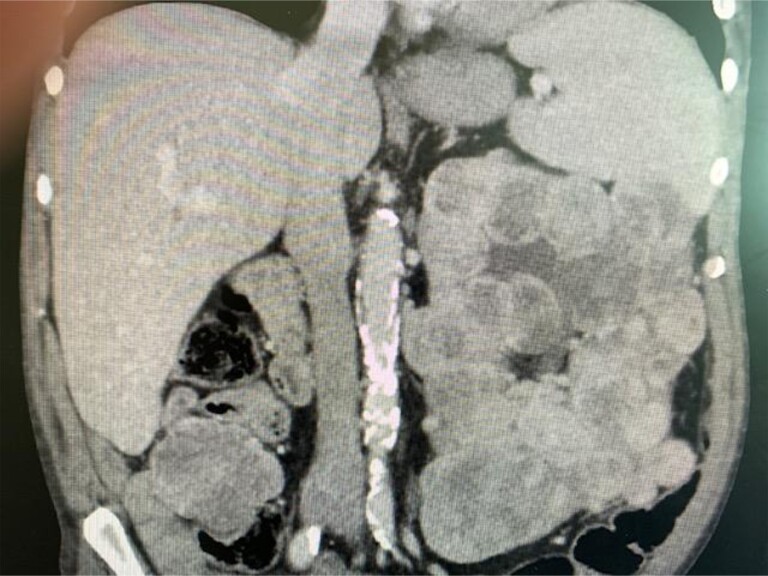
CT imaging of the abdomen and pelvis obtained after the administration of intravenous contrast material revealed a 5 × 5.4 × 18.2-cm lobulated mass infiltrating the left kidney, which was thought to be the putative source of IGF-2 excess.

CT scan of abdomen and pelvis in April 2022 revealed that the heterogeneous hypo-enhancing mass entirely replacing the left kidney now measured 11.6 × 10 × 28 cm (Fig. 2). Given that this mass had not been locally treated, it was considered the putative source of IGF-2 excess. Since surgery and ethanol ablation were deemed to be excessively risky, radiation therapy was recommended. He underwent a course of external radiotherapy to the left renal mass (30 Gy in 10 sessions).

### Outcome and Follow-up

**Figure 3.**
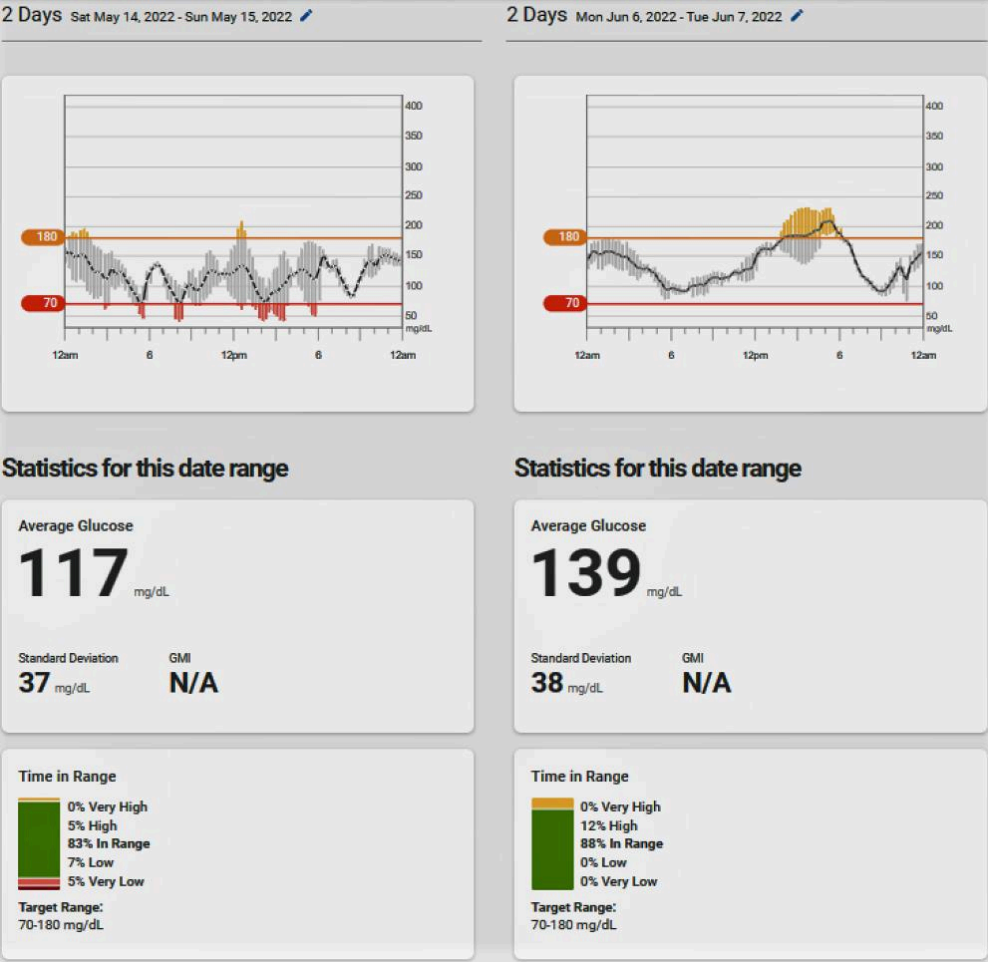
Dexcom G6 blood glucose reading data immediately before and 11 days after completion of palliative XRT.

By day 11 after finishing radiotherapy, the Dexcom G6 blood glucose readings showed that the hypoglycemia had abated and that postprandial hyperglycemia in the diabetic range was becoming evident (Fig. 3). The diazoxide was discontinued and the prednisone was tapered down to 10 mg per day over the next several weeks.

## Discussion

We describe a long-standing type 2 diabetic man diagnosed with a meningeal SFT 24 years ago, found to have metastatic extracranial disease and NICTH, 12 and 23 years later, respectively, despite having stopped insulin therapy 3 years ago. In retrospect, he probably started having unrecognized hypoglycemia at least 1.5 years before his hypoglycemia diagnosis, as suggested by his HbA1c of <4.2% in May 2020.

**Figure 4.**
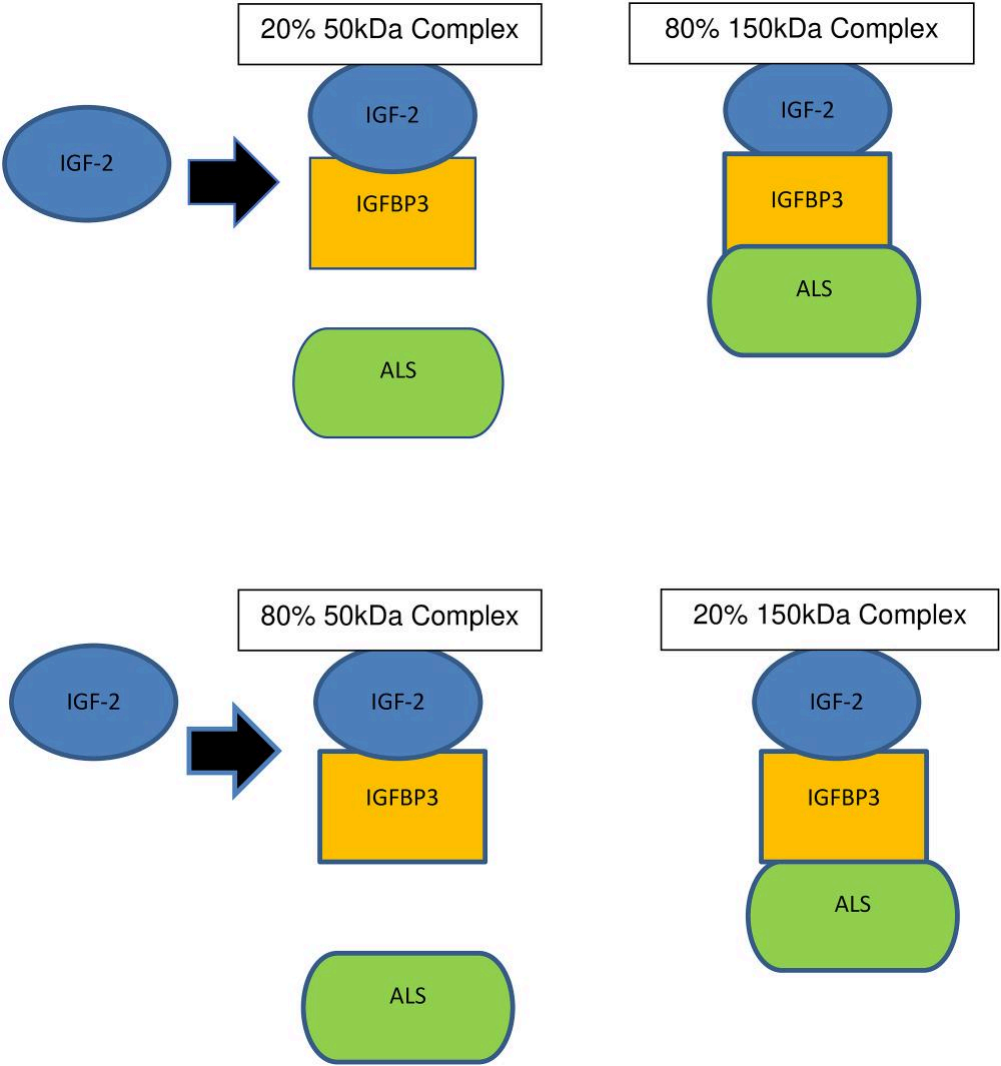
Normal interaction of IGF-2 with binding proteins IGFBP-3 and ALS (modified from reference [1]). In NICTH, there is overproduction of IGF-2 by the tumor and an increased percentage of the 50 kDA binary complex protein which is smaller causing greater vascular permeability and increased insulin-like effects.

IGF-2 is synthesized, under normal circumstances, by the liver, and in patients with NICTH, by certain mesenchymal and epithelial tumors. The precursor of IGF-2 is pre-pro-IGF-2, a 180-amino acid peptide constituted by a 24-amino acid N-terminus, a 67-amino acid IGF-2, and an 89-amino acid C-terminus called the E-domain. The cleavage of the 24-amino acid peptide from the N-terminus originates the pro-IGF-2, a 156-amino acid molecule, and the subsequent removal of the E-domain from the C-terminus gives rise to the “mature” IGF-2 [1]. The IGF-2 (7.5 kDa) binds with IGFBP-3 (40 kDa) to fabricate an approximately 50 kDa binary complex, which can subsequently bind with the acid-labile subunit (ALS) (85 kDa) to produce a 140 to 150 kDa ternary complex. In the normal state, 20% of the “mature” IGF-2 circulates in the binary complex and 80% in the ternary complex [1] (Fig. 4).

In IGF-2-omas, the E-domain, which is underglycosylated, undergoes proteolysis, resulting in an 87-amino acid peptide, one of the “big” IGF-2 molecules, which in conjunction with the increased amount of circulating free “mature” IGF-2, are responsible for the hypoglycemia [1]. In patients with NICTH, decreased hepatic synthesis of ALS and steric interference in the binding of the pro-IGF-2 and IGFBP-3 complexes (60 kDa) to ALS contribute to the predominance of the binary complexes, which can easily exit the vascular space and bind to the insulin receptors, on different tissues to produce the insulin-like effects of IGF-2 [1] including inhibiting the synthesis of GH by the somatotroph; IGF-1, IGFBP-3, and ALS by the liver; and insulin and glucagon by the pancreas [4]. All these changes lead to inhibition of glycogenolysis, gluconeogenesis, ketogenesis, and lipolysis, with the subsequent exhaustion of available glucose and ensuing hypoglycemia.

SFTs can arise nearly anywhere in the body, originating most often in serosal membranes such as the pleura and peritoneum, the dura, and soft tissues. They are typically well-circumscribed and can become large and pedunculated. SFTs can vary in cellularity, which is composed of atypical, but uniform fibroblastic spindle cells in random oriented fascicles (the “patternless pattern”) and storiform arrangements [2]. The behavior of SFTs is difficult to predict based upon histologic examination and for unknown reasons, meningeal SFTs are particularly prone to malignant behavior. An adverse outcome is correlated with the mitotic rate and a risk assessment model has been developed that incorporates patient age, tumor size, mitotic activity, and presence of necrosis [5]. By immunohistochemistry, SFTs are generally CD34 (+), CD99 (+), BCL2 (+), and STAT6 (+). Many SFTs were formally classified as hemangiopericytomas until it was recognized that the latter have the SFT-defining NAB2-STAT6 gene fusion [6].

To our knowledge, only a few cases of paraneoplastic hypoglycemia associated with metastatic meningeal SFTs have been reported [7]. Although the mean time elapsed between onset of NICTH and tumor detection was 8.5 ± 1.9 months in one series [8], the appearance of NICTH many years after the original diagnosis has been rarely documented. Kaneko et al described a patient whose hypoglycemia first manifested 17 years after he was found to have an intraabdominal SFT [9]. Our patient's hypoglycemia was diagnosed 23 years after initial presentation and 12 years after extracranial metastatic disease was first detected.

A PubMed search for cases of *non-islet cell tumor hypoglycemia* identified 208 cases, of which 68 were SFTs/hemangiopericytomas, and of these 34 were characterized as malignant. In this review, other tumors that caused NICTH included 19 gastrointestinal stromal tumors, 19 hepatocellular carcinomas, and 9 breast phylloides tumors, but NICTH was reported with various carcinomas, sarcomas, and lymphomas. There were no reports of NICTH occurring with melanoma. Also, of these 208 cases of NICTH, in 47 cases, the NICTH occurred after tumor diagnosis with a latency of up to 30 years. This may relate to a necessary tumor volume to effect hypoglycemia or perhaps a late genetic aberration. There were 3 cases of meningeal malignant SFTs and all developed NICTH >10 years after initial diagnosis. This search emphasizes that NICTH is associated with a variety of tumors and time periods after diagnosis and that NICTH is often a harbinger of a new cancer or cancer spread indicating the need for further assessment.

Like our patient, other case reports have documented significant improvement or complete resolution of the pre-existing diabetic state and continuous glucose monitoring with CGMs has been used in patients with NICTH to facilitate the management of the patient's hypoglycemia [10].

The mainstay of treatment is complete surgical resection to eliminate the source of total IGF-2, including the “big” and “mature” forms. If this is not possible, surgical tumor debulking to facilitate the treatment of hypoglycemia as well as local antitumor therapy, embolization, chemotherapy, and radiation therapy have also been used. Before and after these more definitive therapies are implemented, a garden variety of medical treatments have been tried to palliate the hypoglycemia, including oral and intravenous glucose, glucagon, diazoxide, octreotide, glucocorticoids, and supraphysiological doses of recombinant human GH [10].

In summary, we describe a patient with NICTH with several particularities, including amelioration of his insulin-requiring type 2 diabetes mellitus followed by fasting hypoglycemia more than 2 decades after his meningeal SFT was originally detected, normal IGF-2 level, IGF-2/IGF-1 ratio < 10, rapid improvement in hypoglycemia after radiation therapy, and management with continuous glucose monitoring.

## Learning Points

- NICTH is a rare paraneoplastic syndrome caused by certain mesenchymal and epithelial tumors. The most common mesenchymal tumors associated with this condition are pleural or extrapleural SFTs, which may be benign or malignant. NICTH indicates a need to search for a causative tumor or newly metastatic cancer.
- The resultant hypoglycemia is produced by the insulin-like effects of the ectopic “big” IGF-2 and the circulating free “mature” IGF-2 peptide.
- The mainstay of treatment is complete surgical resection to eliminate the excessive source of total IGF-2. If this is not possible, palliative tumor debulking is indicated to facilitate the treatment of hypoglycemia.
- A CGM can facilitate the management of hypoglycemia and offer piece of mind to the patient.

## Data Availability

All data are presented in the manuscript.

## Abbreviations

ALS: acid-labile subunit
CGM: continuous glucose monitor
CT: computed tomography
GH: growth hormone
HbA1c: glycosylated hemoglobin
IGF-2: insulin growth factor 2
IGFBP: IGF binding protein
NICTH: non-islet-cell tumor hypoglycemia
SFT: solitary fibrous tumor
